# Design and Optimization of Pioglitazone Hydrochloride Self-Nanoemulsifying Drug Delivery System (SNEDDS) Incorporated into an Orally Disintegrating Tablet

**DOI:** 10.3390/pharmaceutics14020425

**Published:** 2022-02-16

**Authors:** Mahmoud Teaima, Sandra Hababeh, Mai Khanfar, Fares Alanazi, Doaa Alshora, Mohammed El-Nabrawi

**Affiliations:** 1 Department of Pharmaceutics and Industrial Pharmacy, Faculty of Pharmacy, Cairo University, Cairo 11562, Egypt; sandra.hababeh@outlook.sa (S.H.); Mohamed.elnabarawi@pharma.cu.edu.eg (M.E.-N.); 2 Department of Pharmaceutical Technology, Faculty of Pharmacy, Jordan University of Science and Technology, Irbid 22110, Jordan; mskhanfar@just.edu.jo; 3 Department of Pharmaceutics, College of Pharmacy, King Saud University, Riyadh 11451, Saudi Arabia; afars@ksu.edu.sa (F.A.); dalahora@ksu.edu.sa (D.A.)

**Keywords:** orally disintegrating tablet (ODT), solidified self-emulsifying drug delivery system (S-SNEDDS), Pioglitazone Hydrochloride (PGZ), Syloid (SYL), Prosolv

## Abstract

Pioglitazone Hydrochloride (PGZ) suffers from poor aqueous solubility. The aim of this research was to design orally disintegrating tablets with self-nanoemulsifying properties (T-SNEDDS) to improve the Pioglitazone solubility and dissolution rate. Three liquid self-nanoemulsifying systems (L-SNEDDS) were formulated and evaluated for transmittance percentage, emulsification time, particle size, Poly dispersity index (PDI), percentage of content, solubility and stability. The optimum L-SNEDDS formula was converted to a solidified self-nanoemulsifying drug delivery system (S-SNEDDS) by adsorption on Syloid (SYL). Powder characterization tests, such as flowability tests, differential scanning calorimetry (DSC), Fourier transform infrared spectroscopy (FTIR), X-ray diffraction (XRD) and scanning electron microscopy (SEM), were performed for the selected S-SNEDDS formulation. Orally disintegrating tablets (ODT) were formulated by blending S-SNEDDS with tableting excipients. The ODT tablet batch composed of Prosolv was selected for tablet quality control tests, such as hardness, friability, disintegration time, content uniformity, weight variation, in vitro release, in vivo studies and accelerated stability studies. ODT tablets showed accepted mechanical properties and rapid disintegration time (<30 s). No drug degradation was observed at 3 months into the accelerated stability study. The optimized L-SNEDDS, S-SNEDDS and ODT (T-SNEDDS), showed significant enhancement of PGZ in vitro dissolution profiles compared to the pure drug (*p* > 0.05). In vivo pharmacokinetic and pharmacodynamic evaluation of ODTs showed better behavior compared to the raw drug suspension and the commercial tablet (*p* > 0.05). Orally disintegrating tablets revealed a promising potential to improve Pioglitazone poor aqueous solubility, dissolution profile and bioavailability.

## 1. Introduction

Pioglitazone HCl (PGZ) is an antidiabetic drug with an insulin-sensitizing effect. It binds to peroxisome proliferator-activated receptor gamma (PPAR-γ) that increases the transcription of insulin-responsive genes, thus improves tissue sensitivity towards insulin. PGZ is classified as class II under the biopharmaceutics classification system (BCS), meaning it is poorly soluble and highly permeable. Its poor aqueous solubility (0.015 mg/mL) has been a major constrain that hinders its dissolution rate, resulting in therapeutic level failure [1].

Liquid self-emulsifying drug delivery systems (L-SNEDDS) have recently gained importance in the field of pharmaceutical technology for enhancement of solubility and bioavailability of orally administered poorly water-soluble drugs (PWSDs). They are isotropic mixtures of an oil, a surfactant and a cosurfactant [2]. Their potential to enhance the bioavailability is due to their ability to spontaneously form a stable oil-in-water (*o*/*w*) emulsion once agitated with the aqueous phase at a low energy requirement. Upon oral administration, the stomach’s digestive motility provides the necessary agitation for self-emulsification, resulting in a nanosized emulsion (20–200 nm) with a large interfacial area for drug absorption [3]. However, being liquid in nature may have several problems, such as rancidity, leakage or incompatibility with the capsule shell, drug precipitation during manufacturing or in storage [4]. Additionally, some drugs may undergo chemical degradation caused by L-SNEDDS lipid components [5].

Solidification of L-SNEDDS into S-SNEDDS is a remarkable approach to solve such problems and provides formula stabilization benefits [6]. Porous silica adsorbent powders have been successfully utilized to convert lipid formulations into freely flowing powders; such attempts include Lovastatin silica solid dispersions [7], Clopidogrel loaded solid SNEDDS [8] and Celecoxib silica lipid hybrid microparticles [9]. However, S-SNEDDS may poorly disintegrate with an inclined dissolution rate due to the strong adhesion interaction between lipid components and the adsorbent particles [10]. In contrast to conventional tablets, oral disintegrating tablets (ODTs) can offer a distinctive advantage due to their rapid disintegration in a matter of seconds once they come in contact with the saliva. They are also more patient compliant to geriatric, bedridden and psychologically ill patients [11]. However, no previous works have been published in regard to PGZ orally disintegrating tablets with self-nanoemulsifying properties. Accordingly, the present study aims to develop PGZ orally disintegrating tablets (T-SNEDDS) derived from L-SNEDDS to enhance poor drug solubility and L-SNEDDS formulation stability. First, the drug was incorporated into a liquid formulation (L-SNEDDS) using a screened oil, a surfactant and a cosurfactant. Second, it was converted to solidified formulation (S-SNEDDS) by adsorption onto Syloid^®^244 FP. Last, S-SNEDDS was further formulated into orally disintegrating tablets (T-SNEDDS) that have a self-nanoemulsifying nature in aqueous gastrointestinal media. The optimized tablets were evaluated by tablet quality control tests, in vitro dissolution, in vivo studies and stability studies.

## 2. Materials and Methods

### 2.1. Materials

Pioglitazone HCl (PGZ) (purity > 99.5%) was obtained by Riyadh Pharma manufacturer (Riyadh, Saudi Arabia). Capryol^TM^ 90, Transcutol^TM^ HP, Labrafil^®^M1944CS and Labrasol^®^ were purchased from Gattefossé (Lyon, France). Capmul MCM was obtained by Abitec (Columbus, OH, USA). Olive oil and Soybean oil were gifted from Croda (East Yorkashire, UK). Tween^®^ 80 and Oleic acid were acquired from Sigma–Aldrich (St. Louis, MO, USA). Propylene glycol (PG) was obtained from Winlab (Gemini-house, UK). Cremophor^®^ EL and Cremophor^®^ RH 40 were supplied from BASF (Ludwigshafen, Germany). Pure gelatin capsules (size 000) were supplied by the Center of Applied Research and Advanced Studies, Faculty of Pharmacy, Cairo University (CARAS). Silica powders, Aerosil 200^®^, Syloid^®^ 244 FP (SYL), Neusilin^®^ grades US2 and UFL2 were donated from Fuji Chemical Industries (Osaka, Japan). Prosolv^®^ ODT G2, mannitol, fructose and crospovidone were gifted by GRS pharma (Rosenberg, Germany). All other chemicals were of analytical grade.

### 2.2. Design of PGZ Liquid Self-Nanoemulsifying Drug Delivery Systems (L-SNEDDS)

#### 2.2.1. PGZ Equilibrium Solubility Study

The saturated equilibrium solubility of PGZ was examined in different oils (Campul MCM, Oleic acid, Olive oil, Soybean oil and Caproyl 90), surfactants (Cremophor^®^ RH40, Labrafil^®^ M1944CS, Labrasol^®^, Tween^®^80 and Cremophore^®^ EL), and cosurfactants (Transcutol HP and Propylene glycol (PG)). According to the shake flask method described by Higuchi and Connors [12], an excess known amount of PGZ (100–500 mg) was added to 2 mL of different oils, surfactants and cosurfactants, the mixture was vortexed for 2 min, and placed in a shaker water bath (SW22 Julabo, Labortechnik GmbH, Germany) at 100 rpm and 37 ± 0.5 °C for 72 h to attain equilibrium. Then, the equilibrated samples were centrifuged at 4000 rpm for 10 min. Each supernatant was collected, filtered by 0.45 µm syringe filter, then diluted with methanol for spectrophotometric quantitative determination via a UV-validated method using UV-visible spectrophotometer (Libra S22, Biochrom, Cambridge, UK) at 269 nm. Each experiment was performed in triplicate and the standard deviation (SD) was calculated. The results are illustrated in Table 1.

#### 2.2.2. Construction of Pseudo-Ternary Phase Diagrams

**Figure 1:**
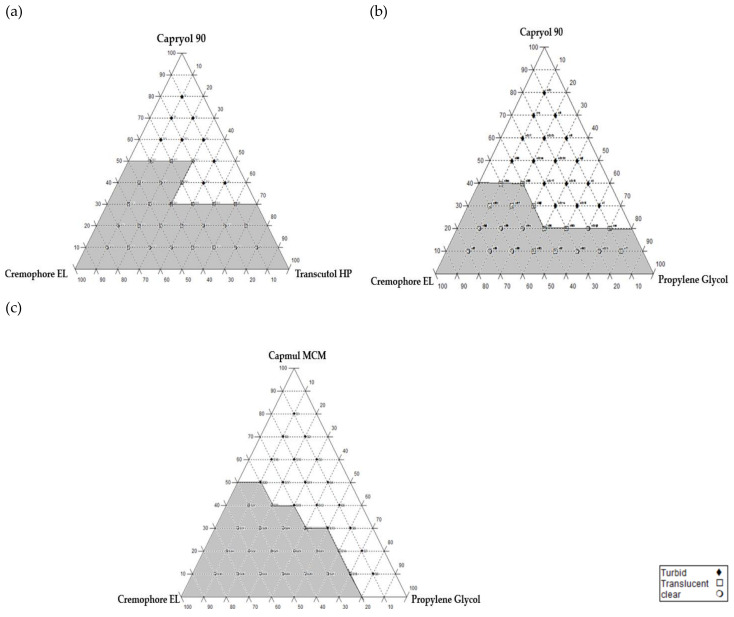
Pseudo-ternary phase diagrams for (**a**) system I (Capryol/Cremophor EL/Transcutol HP), (**b**) system II (Capryol/Cremophor EL/Propylene glycol) and (**c**) system III (Capmul MCM/Cremophor EL/Propylene glycol).

Components utilized to construct the pseudo-ternary phase diagrams were chosen based on solubility study results. Accordingly, three phase diagrams were constructed using Caproyl 90 and Campul MCM as oily phases, Cremophore^®^ EL as a surfactant, Transcutol HP and Propylene glycol (PG) as cosurfactants. For each selected SNEDDS system, oil/surfactant/cosurfactant were prepared at 36 predetermined ratios with a 10% interval for a total concentration of 100% *w*/*w* [13]. At each of the 36 prepared systems, 1 gm (oil/SA/Cos) was vortexed, diluted with 200 mL deionized water and magnetically stirred at 100 rpm at 37 ± 0.5 °C. Immediate visual inspection against strong light was carried out to evaluate spontaneous emulsification, clarity of the solution and nano-emulsion formation process. The clarity of the resulted solutions was confirmed by measuring the percentage of transmittance (% T) of each prepared mixture using UV spectrophotometer at 638 nm. Then, the diluted emulsions were left for 24 h for stability assessment. Clear (% T ≥ 95%) and translucent or slightly bluish (% T ≥ 90%) emulsions were identified as grade A and B emulsions, respectively [14,15]. These emulsions were considered to draw the phase diagram. Figure 1 shows the three-phase diagrams drawn using CHEMIX ternary plot software (CHEMIX School Ver. 3.60, Pub. Arne Standnes, Bergen, Norway).

#### 2.2.3. Preparation of PGZ L-SNEDDS

Twelve formulations were selected from each ternary phase plot, four from each system (I, II and III). For each 1 mL formula, 15 mg PGZ was dissolved in the accurately weighed amount of oil and cosurfactant, vortexed, then heated at 50 °C until the mixture became transparent, the mixture was left to cool down, then, the weighed amount of surfactant was further added and vortexed to obtain the final L-SNEDDS formulations. The compositions of PGZ loaded selected liquid self-nanoemulsifying formulations (L-SNEDDS) are shown in Table 2.

#### 2.2.4. Assessment of PGZ L-SNEDDS

##### Visual Inspection, Self-Emulsification Time and Robustness

Selected SNEDDS preconcentrates were visually evaluated for their clarity after dilution and the percentage of transmittance was assessed spectrophotometrically at 638 nm using deionized water as a blank. Then, the time required for nano emulsion formation was recorded at 200 times dilution with 0.3 M HCl buffer solution under constant stirring at 100 rpm at 37 ± 0.5 °C [16]. Later, the diluted solutions were stored for 24 h and observed for phase separation. Systems that were stable against separation or drug precipitation were considered to be “robust to dilution” [17,18].

##### Droplet Size (PS) and Polydispersity Index (PDI)

Droplet size and polydispersity index (PDI) analysis were performed using Malvern Zetasizer (Malvern Instruments, Malvern, UK) at 25 °C. Droplet size is crucial for self-emulsification, it affects the rate and extent of drug release, and is a good indication for an emulsion’s physical stability. PDI is an indication for uniformity of size distribution within the formula. The higher PDI values, the lower uniformity of the formulation, while closer to zero values indicate more homogenous emulsions. All samples were sonicated prior to droplet size and PDI determination.

##### Percentage of Content

PGZ loaded SNEDDS formulations equivalent to 15 mg PGZ were tested for their content. 0.1 gm of each preparation was diluted to 25 mL methanol, vortexed, then analyzed spectrophotometrically at 269 nm using methanol as a blank.

##### L-SNEDDS In Vitro Release

In vitro release studies were performed according to FDA specifications using the United States Pharmacopeia (USP) dissolution apparatus type II (model: PT DT70, Pharma test, Hainburg, Germany) coupled with a paddle stirrer at 75 rpm in 0.3 M HCl at 37 ± 0.5 °C (pH = 2) [19]. One gram of each L-SNEDDS formulation equivalent to 15 mg PGZ was filled in a soft gelatin capsule (size 000) and the release was conducted in 900 mL buffer for one hour. 5 mL samples were withdrawn at a predetermined time intervals of 5, 10, 15, 30, 45 and 60 min, filtered using 0.45 µm syringe filter, replaced by fresh buffer and analyzed by UV at 269 nm. Each experiment was carried out in triplicate.

##### L-SNEDDS Equilibrium Solubility

In order to assess the maximum PGZ loading ability of selected L-SNEDDS in systems I, II and III, a solubility experiment was performed. An excess amount of PGZ (100–500 mg) was added to one gram of each L-SNEDDS formulation. Mixtures were vortexed then placed in a shaker water bath adjusted at 37 ± 0.5 °C for 72 h. After equilibrium, mixtures were centrifuged at 4000 rpm for 10 min. The supernatant was then collected and measured spectrophotometrically after appropriate dilutions with methanol at 269 nm; results are shown in Table 3.

##### Thermodynamic Stability

A three-phase thermodynamic stability test was conducted for L-SNEDDS formulations. The phase 1 test included three hearting-cooling cycles carried between 4 °C (refrigerator) and 50 °C (Oven) with storage at each temperature for 48 h. Passed formulations were then subjected to a phase 2 test, where centrifugation of formulations was carried out at 3500 rpm, 25 °C for 30 min. Formulations that did not show phase separation were subjected to a phase 3 test that included the storage of formulations at three freeze/thaw cycles between −21 °C (freeze) and +25 °C (thaw) for not less than 48 h [20]. Results are shown in Table 4.

### 2.3. Preparation and Characterization of Solidified Self-Nanoemulsifying Formulations (S-SNEDDS)

One gram of the optimized L-SNEDDS formula was added in a drop-wise manner over four types of adsorbent powders (Neusilin^®^ US2, Neusilin^®^ UFL2, Syloid^®^ 244FP, Aerosil^®^ PH200) with a ratio of 1:0.5 (*w*/*w*) (L-SNEDDS: adsorbent) as shown in Table 5. Mixtures were homogenized using a glass rod for 3 min to ensure uniform distribution. The influence of adsorbent type on the formula dissolution profile was investigated using the United States Pharmacopeia (USP) dissolution apparatus type II (model: PT DT70, Pharma test, Hainburg, Germany) in 900 mL 0.3 M HCl (pH = 2) buffer for 120 min. The adsorbent powder with the highest dissolution profile was selected for further characterization and tablet formulation. 

#### 2.3.1. Determination of Optimum L-SNEDDS: Adsorbent Ratio 

L-SNEDDS formulation was mixed at ratios of 1:0.5, 1:1 and 1:2 (*w*/*w*) with the selected adsorbent Syloid (SYL). Then, powder flowability tests were performed for determination of powder micrometric properties, such as compressibility index (CI), Hausner ratio (HR) and angle of repose (θ) (Table 6). 

#### 2.3.2. Differential Scanning Calorimetry (DSC)

For thermotropic properties assessment, thermal analysis was carried out using Shimadzu differential scanning calorimeter (DSC-50, Kyoto, Japan). Samples of 3–5 mg were heated in aluminum pans at a scanning rate of 10 °C/min under dry nitrogen flow (20 mL/min) and over a temperature range of 0–230 °C. Empty aluminum pans were used as references. Differential scanning calorimetry (DSC) thermograms were recorded for pure PGZ, pure SYL and the S-SNEDDS formulation.

#### 2.3.3. Fourier Transform Infrared Spectroscopy (FT-IR)

Fourier transform infrared (FTIR) spectroscopy was recorded for the characterization of crystallinity and polymorphic changes using a Shimadzu FTIR- 8400 spectrophotometer, Japan. FTIR Spectrum of pure PGZ was compared to the S-SNEDDS formulation. Samples were blended with potassium bromide powder. The test was conducted over a frequency range of 4000–400 with a resolution of 4 cm^−1^ and the average of 16 scans was recorded.

#### 2.3.4. X-ray Diffraction (XRD)

Using an Ultima IV X-ray diffractometer (Rigaku, Tokyo, Japan), powder X-ray diffraction (PXRD) patterns of pure PGZ, pure SYL and the S-SNEDDS formulation were obtained for additional detection of crystallinity and polymorphic changes. Samples were analyzed using cobalt radiation (CuKa) at a voltage of 40 kV and a current of 40 mA. The scan step was 0.02° with a 2θ range of 5–70° at angular increment of 4 degrees/minute.

#### 2.3.5. Scanning Electron Microscopy (SEM)

Surface morphology was visualized using a Philips’s scanning electron microscope (model FEI Quanta FEG 450, Amsterdam, Netherlands). Samples were fixed on an aluminum stub by a double-sided sticky disc of conductive carbon, then coated with a thin gold pallidum layer. The electron beam was scanned over the specimen to produce a digital image for pure SYL and S-SNEDDS formulation.

### 2.4. Preparation and Characterization of Orally Disintegrating Tablets (ODT)

S-SNEDDS powder containing SYL was blended with different tableting excipients; all ingredients were sieved and blended, then, compressed at a compression force of 6 kN (Kilonewton) using Shimadzu IR hydraulic press machine (Shimadzu Hydraulic Equipment, Kyoto, Japan). Hardness of produced tablets was measured at each compression process. The composition of three prepared ODT batches is shown in Table 7.

#### 2.4.1. Hardness, Friability and Disintegration Testing

The force needed to break a tablet was measured using the Copley Hardness Tester, Switzerland. For friability testing, 20 tablets were weighed accurately (Wi) and rotated using Erweka friabilator (Langen, Germany) for 4 min at a 25 rpm rotation speed. Then, tablets were weighed after performing the test (Wa). The friability percentage was calculated according to Equation (1). Another six tablets were placed individually in the baskets of the disintegration tester (Erweka, Germany) containing the dissolution medium at 37 ± 0.5 °C. The time required for a tablet to pass totally through the basket mesh was recorded.

Weight loss % = ((Wi − Wa)/Wi) × 100(1)

#### 2.4.2. Content Uniformity and Weight Variation

In order to measure content uniformity, 10 tablets were crushed individually, dissolved in methanol, centrifuged, and filtered using a 0.45 µm syringe filter, then analyzed spectrophotometrically at 269 nm. Tablet weight variation was determined by weighing 10 tablets individually; the percentage of weight variation was calculated by dividing each tablet weight over the average weight of the 10 tablets.

#### 2.4.3. In Vitro Release Study

In vitro release for the selected ODT batch was tested in triplicate using the United States (USP) dissolution apparatus type II (model: PT DT70, Pharma test, Hainburg, Germany) at 37 ± 0.5 °C. Each ODT was placed in 900 mL 0.3 M HCl buffer (pH = 2). The paddle rotation speed was adjusted to 75 rpm. 5 mL samples were withdrawn at predetermined time intervals of 5, 10, 15, 30, 45, 60 and 120 min, collected, filtered using 0.45 µm syringe filter, replaced by fresh buffer and analyzed spectrophotometrically at 269 nm. 

#### 2.4.4. Pharmacokinetic Study in Healthy Rats

Twenty-four Wistar Albino male rats (200 ± 10 gm) were divided into four groups (*n* = 6); Group I (commercial product Actos^®^), Group II (ODT), Group III (control) and Group IV (raw PGZ). Orally administered dose was equivalent to 30 mg/kg dissolved in 10% DMSO for the three groups (I, II and IV) calculated upon a rat’s individual weight to avoid any possible toxicity. Approximately 0.5 mL blood samples were collected from rat tail veins into lithium heparinized test tubes at 0, 1, 2, 3, 4, 5, 6, 12 and 24 h post dose. Plasma was obtained by centrifugation at 14,000 rpm for 15 min. Then samples were stored in deep freezer at −80 °C. Sample analysis was performed according to the validated UPLC-MS/MS analytical method reported by Imran et, al [21]. UHPLC Thermo Dionex (Ultimate 3000, Idstein Germany) UV- VIS detector controlled with Chromeleon 7.2 software and equipped with C18 column 150 × 4.6 mm 5 µm (HiQSil KYA, Tokyo, Japan) was utilized. The mobile phase consisted of 0.1 mM ammonium acetate, acetonitrile and acetic acid in the ratio of 25:25:1 respectively. The injection volume was 80µL at 1.2 mL/min. The detector was set at 269 nm. Analysis of all pharmacokinetic parameters was performed using PK-Solver add-in program for non-compartmental analysis by Microsoft Excel (Yong Zhang, Nanjing, China) [22].

#### 2.4.5. Pharmacodynamic Study in Diabetic Rats

Twenty-four Wistar Albino male rats (200 ± 10 gm) were divided into four groups (*n* = 6); Group I (commercial product Actos^®^, Arab Pharmaceutical Manufacturing (APM), Amman, Jordan), Group II (ODT), Group III (+ve control) and Group IV (-ve control). Three days before conducting the experiment, diabetes was induced in groups I, II and III by a single intraperitoneal injection of aqueous Streptozocin (STZ) (50 mg/kg) solubilized in phosphate buffer pH 4.5. Blood glucose level was determined by a blood drop from the tail veins of the rats using the Accu-check blood glucose meter. Rats showing serum glucose levels above 200 mg/dL were considered diabetic. Diabetic rats received the calculated dose of the corresponding formula equivalent to 30 mg/kg through an oral gavage needle. Blood glucose level was measured at 1, 2, 4, 6, 12 and 24 h.

#### 2.4.6. Accelerated Stability Studies

Stability studies of the optimized ODT formulation were performed at different temperature conditions according to ICH guidelines at 25 ± 2 °C, 60 ± 5% RH and 40 ± 2 °C, 75 ± 5% RH for 3 months [23]. The physical appearance and the dissolution profile of aged tablets were assessed.

### 2.5. Statistical Analysis

One-way analysis of variance (ANOVA) followed by post-hoc (LSD) tests were performed using SPSS 25 software to interpret significant difference between data obtained by in vitro, in vivo and stability tests; a *p*-value less than 0.05 was considered significant.

## 3. Results

### 3.1. PGZ Solubility Study

Among oil materials, the highest PGZ solubility was attained by Caproyl 90 with a value of 44.69 mg/mL and Capmul MCM with a value of 32.93 mg/mL. Crempohore EL showed the best solubilizing potential among all the tested surfactants with a solubility of 48.38 mg/mL, while Propylene glycol (249.99 mg/mL) followed by Transcutol HP (109.38 mg/mL) showed the highest PGZ solubility as cosurfactants (Table 1).

### 3.2. Construction of Pseudo-Ternary Phase Diagrams 

Figure 1 illustrates the three phase diagrams constructed for System I (Capryol 90/Cremophor EL/Transcutol HP), System II (Capryol 90/Cremophor EL/Propylene glycol) and System III (Capmul MCM/Cremophor EL/Propylene glycol). The shaded area indicted the nanoemulsion region was constructed using only clear (A) and translucent (B) formulations [24]. The emulsification region was the highest in system I > system II > system III.

### 3.3. Assessment of PGZ L-SNEDDS

#### 3.3.1. Visual Inspection, Self-Emulsification Time and Robustness

All selected formulations were clear (graded as A), except, formulations S18-i and S24-ii were bluish and translucent (graded as B) with oil amount of 30% and 40% *w*/*w* respectively (Table 3), while the self-emulsification time ranged from 15 to 60 s regarding to the formula composition. It was noticeable that rapid emulsification contributed to lower oil contents (10–20% *w*/*w*), the same as to higher cosurfactant content (40–60% *w*/*w*) that resulted in mixtures with lower viscosities. However, all formulations were robust to 200 times dilution with 0.3 M HCl buffer; no signs of phase separation and precipitation after 24 h storage were detected.

#### 3.3.2. Droplet Size (PS) and Polydispersity Index (PDI)

The mean droplet size of the formulations was shown in Table 3, the droplet size ranges were for system I formulations from 36.85 nm to 61.82 nm, for system II form 15.58 nm to 52.97 nm, and for system III from 14.12 nm to 349.73 nm. PDI values for the three systems ranged from 0.05 to 0.5 which indicated a good droplet size uniformity. Polydispersity values below 0.6 suggest good uniformity of droplet size distribution [25]. 

#### 3.3.3. Percentage of Content

The maximum and minimum assays for drug content in all prepared L-SNEDDS formulations were 110.13% and 94.28%, respectively, which complies with the USP guidelines acceptance criteria (±15%).

#### 3.3.4. L-SNEDDS Equilibrium Solubility

**Figure 2:**
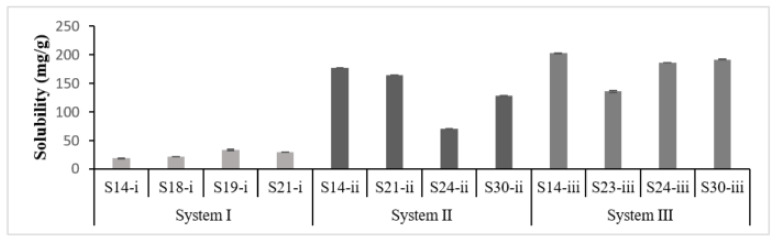
Graphical representation of PGZ equilibrium solubility in selected SNEDDS formulations. S-i, S-ii and S-iii are formulations related to systems I, II and III, respectively.

PGZ solubility in L-SNEDDS reflects the drug loading capacity. In that, a remarkable variability was observed owing to the formulation composition, specifically the type of and the amount of cosurfactant added rather than the type of oil (Figure 2). S14-i (system I) showed the least PGZ solubility of 18.47 mg/g with 60% Transcutol, while S14-iii (system III) showed the highest PGZ solubility of 202.56 mg/g with 60% Propylene glycol.

#### 3.3.5. Thermodynamic Stability

Results revealed that the oil concentration plays an important role in the stability of the formulation; the lesser oil content, the more stable the formulation was. It was noticed that formulations with oil content ranging from 10–20% could pass the three phase stability studies, such as S14-i, S14-ii, S14-iii and S30-iii, whereas failed formulations contained higher oil content ranging from 30–40%, such as S18-i, S24-ii and S23-iii (Table 4).

The selection of optimum L-SNEDDS for solidification was performed by keeping a good balance between the low droplet size (<200 nm), high drug loading capacity, accepted stability and enhanced release profile [26]. Accordingly, S14-iii formulation was utilized in a further solidification process.

### 3.4. Optimization and Characterization of Solidified Self-Nanoemulsifying Formulations (S-SNEDDS)

According to the in vitro release results obtained by S-SNEDDS formulations containing four different types of adsorbents a shown in Section 3.5.2, SNEDDS-3 composed of Syloid^®^244FP (SYL) was preferred over the other formulations to be utilized in further ODT tablet formulation.

#### 3.4.1. Micrometric Properties for Determination of Optimum L-SNEDDS: Adsorbent Ratio

As shown in Table 6, R1 showed high caking and wet powder, upon increasing the percentage of SYL, the caking was reduced along with improved powder flowability. Hence, R4 showed excellent powder flowability (CI < 10, HR = 1–1.11, θ= 25–30), also, R3 showed a fine dry powder with good flowability (CI = 11–15, HR = 1.12–1.18, θ = 31–35). However, R2 was preferred to R3 and R4 aside from its good flowability because it possess a higher SNEDDS ratio (about 66%), which led to higher drug loading within the final formula. Accordingly, R2 (1:0.5) was selected as the optimum ratio for S-SNEDDS formulation and was utilized in ODT tablet formulation.

#### 3.4.2. Differential Scanning Calorimetry (DSC)

**Figure 3:**
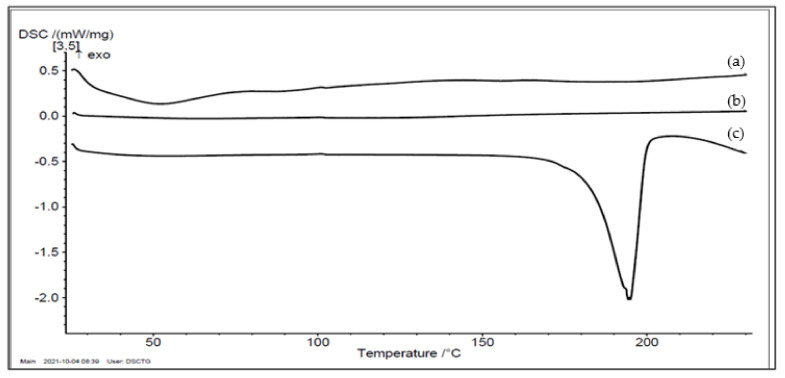
DSC thermograms of (a) PGZ loaded S-SNEDDS, (b) pure SYL and (c) pure PGZ. Abbreviations: DSC, differential scanning calorimetry; SYL, Syloid; S-SNEDDS, solidified self-nanoemulsifying drug delivery system; PGZ, Pioglitazone Hydrochloride.

Pure PGZ exhibited a sharp endothermic peak detected at 197.3 °C, corresponding to its melting point and its crystalline state, while, SYL had no characteristic endothermic peak due to its amorphous state (Figure 3). DSC thermogram of S-SNEDDS showed a complete disappearance of PGZ endothermic peak; this indicated the complete solubilization of the drug within the S-SNEDDS and that the adsorption process was successful in keeping the drug in the solubilized form protected from precipitation [27]. A peak at 53 °C was observed for S-SNEDDS thermogram, corresponding to the dehydration process.

#### 3.4.3. Fourier Transform Infrared Spectroscopy (FT-IR)

**Figure 4:**
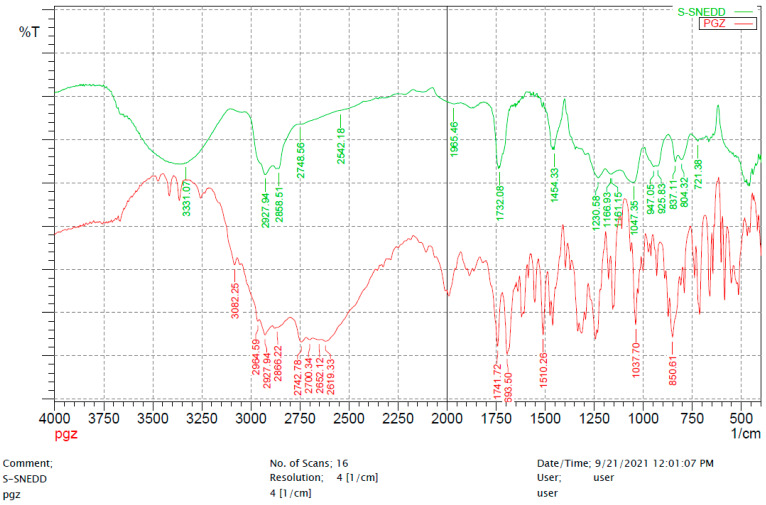
FTIR spectra of (a) PGZ loaded S-SNEDDS, (b) pure PGZ. Abbreviations: FTIR, Fourier-transform infrared spectroscopy; S-SNEDDS, solidified self-nanoemulsifying drug delivery system; PGZ, Pioglitazone Hydrochloride.

PGZ spectrum showed a peak at 3082.89 cm^−1^ for N–H stretching, whereas peaks at 2964.59 cm^−1^ and 2742.66 cm^−1^ represented the stretching of aliphatic C–H structure. A sharp absorption peak was observed at 1741.72 cm^−1^ that may be attributed to carbonyl C=O stretching structure vibration. A peak at 1510.26 cm^−1^ indicated the presence of C=N group and a peak at 693.5 cm^−1^ was attributed to the C–S group. The absence of PGZ characteristic peaks in the S-SNEDDS pattern confirmed that the drug was transformed into an amorphous state, and proves the complete adsorption of L-SNEDDS on the surface of the adsorbent molecules (Figure 4).

#### 3.4.4. X-ray Powder Diffraction (XRPD)

**Figure 5:**
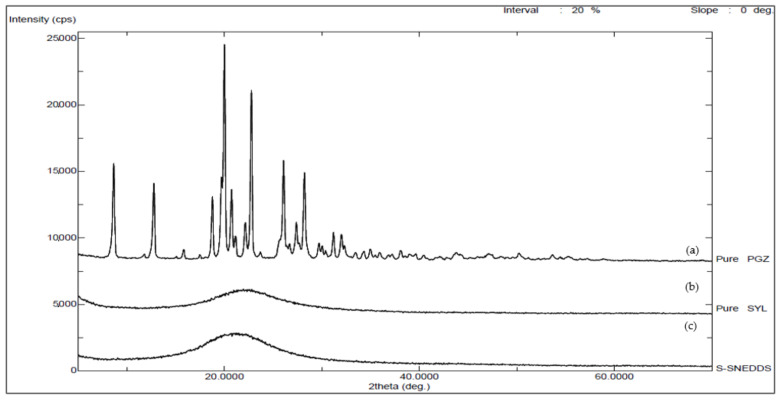
XRPD thermograms of (a) pure PGZ, (b) pure SYL and (c) PGZ loaded S-SNEDDS. Abbreviations: XRPD; X-ray powder diffraction; SYL, Syloid; S-SNEDDS, solidified self-nanoemulsifying drug delivery system; PGZ, Pioglitazone Hydrochloride.

The diffractogram of the pure PGZ exhibited typical sharp peaks at 0–30 (2θ) contributing to its crystalline state. In contrast, XRPD patterns of pure SYL and S-SNEDDS were clearly distinct from that of the pure drug. Characteristic peaks of PGZ were not detectable in PGZ loaded S-SNEDDS, suggesting the conversion to an amorphous state (Figure 5).

#### 3.4.5. Scanning Electron Microscopy (SEM)

**Figure 6:**
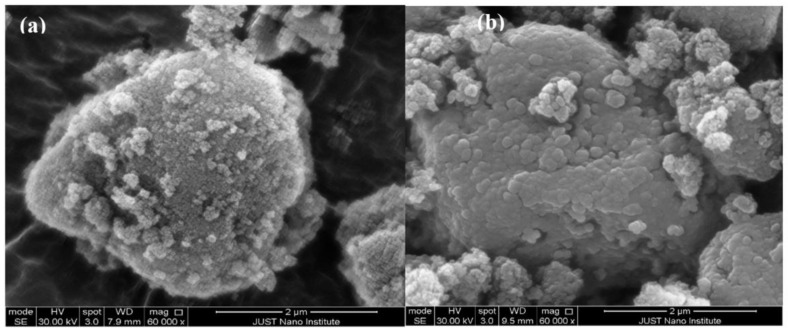
SEM images of (**a**) Pure SYL and (**b**) PGZ loaded S-SNEDDS. Abbreviations: SEM, scanning electron microscopy; SYL, Syloid; S-SNEDDS, solidified self-nanoemulsifying drug delivery system; PGZ, Pioglitazone Hydrochloride.

SEM study indicated that pure SYL showed spherical shaped rough particles with a small particle at the top of its surface (Figure 6a), while the image of PGZ loaded S-SNEDDS showed that the adsorption process caused smoothening of the SYL surface without significant changes in the morphology of the particles (Figure 6b).

### 3.5. Characterization of Oral Disintegrating SNEDDS Tablets (T-SNEDDS)

Three ODT tablet batches were formulated: ODT1, ODT2 and ODT3 (Table 7). ODT1 consisted of Prosolv coprocessed excipient, while ODT2 and ODT3 consisted of acdisol, lactose and acdisol and mannitol, respectively. Only the ODT1 batch was considered as ODT2 and ODT-3 tablets were insufficiently hard (hardness 1–2 kp). Thus, ODT-1 batch tablets were considered in further physicochemical characterization.

#### 3.5.1. Tablet Quality Control Assessment 

The optimized ODT tablets showed weight variation, hardness, friability, percentage of content and disintegration time within the official limits (Table 8). All tablets expressed a percentage of weight variation within the pharmacopeial limits (±5) of tablet weight. Moreover, they possessed good mechanical strength with sufficient hardness of 5.73 ± 0.63 kp and a friability percentage of 0.68% [28,29]. The tablets rapidly disintegrated with an average disintegration time of 28.36 ± 0.95 that is less than 30 s, which meets the ODT tablet criteria [30,31].

#### 3.5.2. In Vitro Release Studies

##### In Vitro Release of Liquid SNEDDS (L-SNEDDS)

**Figure 7:**
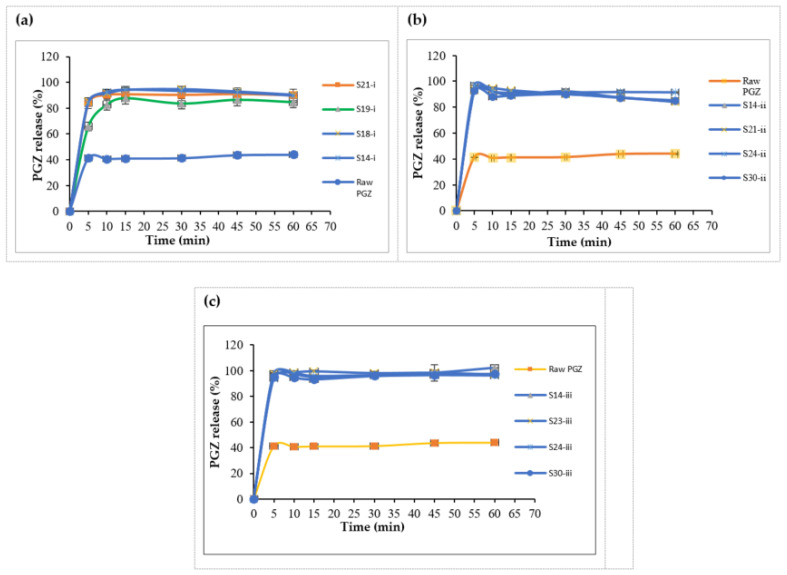
In vitro dissolution profile of PGZ from L-SNEDDS formulae: (**a**) System I, (**b**) System II and (**c**) System III. Abbreviations: L-SNEDDS, liquid self-nanoemulsifying drug delivery system; PGZ, Pioglitazone Hydrochloride.

Raw PGZ showed poor dissolution with 44% maximum release within 60 min run time, while all prepared liquid SNEDDS formulations had shown similar immediate release patterns of >80% after 10 min (Figure 7a–c). Accordingly, the release of the drug was significantly enhanced by optimized L-SNEDDS (*p* < 0.05). S14-iii formulation achieved 100% release after 60 min, as the behavior of the dissolution profile is directly proportional to the droplet size, and also to the solubility of the drug in the formulation, S14-iii showed nanosized droplets with the highest solubilizing capacity that would increase the drug absorption and thus bioavailability [32].

##### In Vitro Release of Solidified Formulation (S-SNEDDS)

**Figure 8:**
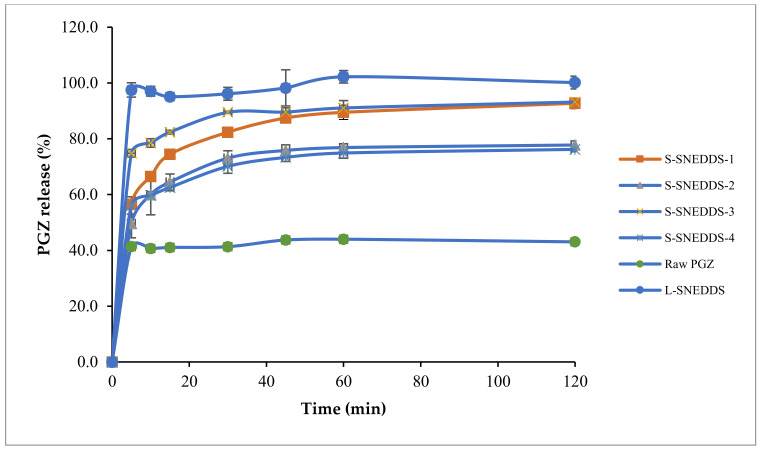
In vitro dissolution profile of PGZ from L-SNEDDS, S-SNEDDS (1 to 4) and raw drug. Abbreviations: S-SNEDDS, solidified self-nanoemulsifying drug delivery system; L-SNEDDS, liquid self-nanoemulsifying drug delivery system; PGZ, Pioglitazone Hydrochloride.

The percentage of drug release from S-SNEDDS-2 and S-SNEDDS-4 with a release of 77.8% and 76.2% over 120 min run time, those formulations composed of Neusilin^®^ UFL2 and Aerosil^®^ PH200, respectively among the other solidified formulations. However, the release was 92.7% attained by S-SNEDDS-1 composed of Neusilin US2^®^ and 93.2% obtained by S-SNEDDS-3 composed of Syloid^®^ 244FP, over 120 min run time (Figure 8). Hence, S-SNEDDS-1 and S-SNEDDS-3 release profile did not differ significantly when compared to the liquid formulation (S14-iii) (*p* > 0.05).

##### In Vitro Release of Orally Disintegrating Tablets (T-SNEDDS)

**Figure 9:**
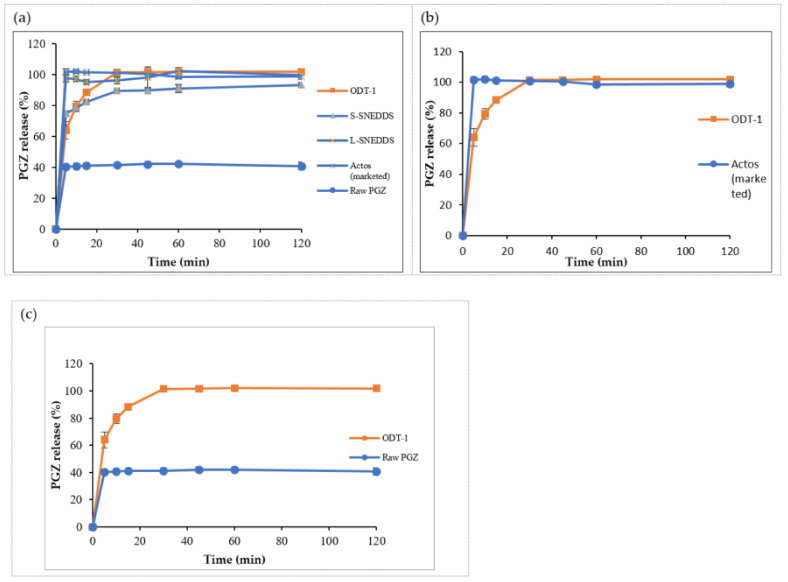
In vitro dissolution profile of: (**a**) PGZ from the optimized ODT batch, S-SNEDDS, L-SNEDDS, commercial tablet and raw PGZ, ODT versus marketed tablet (**b**) and ODT versus raw drug (**c**). Abbreviations: L-SNEDDS, liquid self-nanoemulsifying drug delivery system; S-SNEDDS, solidified self-nanoemulsifying drug delivery system; ODT, oral disintegrating tablet; PGZ, Pioglitazone Hydrochloride.

ODT tablets showed a significant higher release than the solidified S-SNEDDS formulation (*p* < 0.05) (Figure 9a). In addition, the ODT release pattern did not differ significantly from the release of the marketed tablet (Figure 9b) (*p* > 0.05). However, the ODT tablet obtained a release of 88% of PGZ after 15 min and reached 100% after 30 min, which proved the solubility enhancement of PGZ by self-nanoemulsifying properties by a 2.5-fold increase in the release of raw PGZ (*p* < 0.05) (Figure 9c). 

#### 3.5.3. In Vivo Studies 

##### Pharmacokinetic Study in Healthy Rats

**Figure 10:**
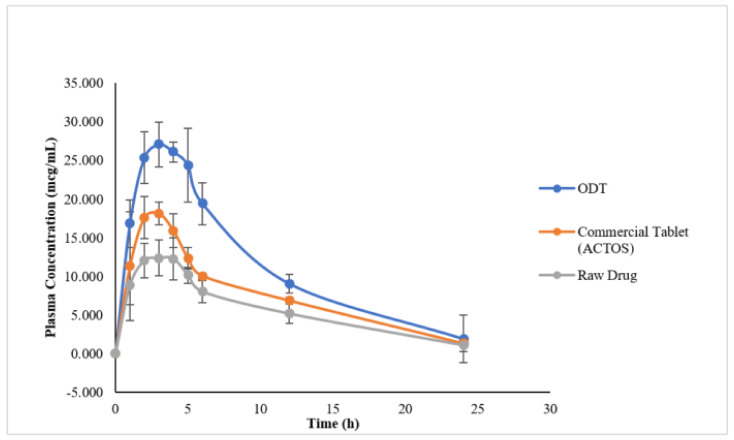
Plasma concentration vs. time profile of ODT tablets, commercial table and raw PGZ after a single dose oral administration to Wistar rats (mean ± SD, *n* = 6). Abbreviations: ODT, orally disintegrating tablet; PGZ, Pioglitazone Hydrochloride.

Drug plasma concentration in rats receiving ODT (T-SNEDDS) formulation was shown to be significantly higher (*p* < 0.05) than in those treated not only with the raw drug suspension, but also the commercial product (Figure 10). Maximum drug plasma concentration (C_max_) after about 3 h for ODT formulation was 27.77 ± 2.55 µg/mL as compared to 17.40 ± 3.38 µg/mL and 12.91 ± 2.24 µg/mL for the commercial product and raw drug suspension, respectively. Corresponding AUC values of ODT formulation showed 1.69- and 2.06-fold increase in the bioavailability as compared to the commercial product and raw drug suspension, respectively (Table 9). There was no significant difference in the mean residence time (MRT) and the time to reach maximum plasma concentration (t_max_) values among the three formulations with values of 7 h and 3 h, respectively (*p* > 0.05).

##### Pharmacodynamic Study in Diabetic Rats

**Figure 11:**
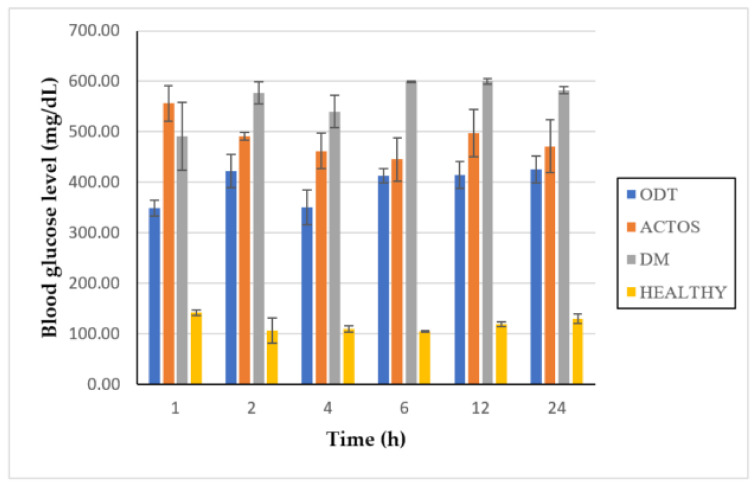
Graphical representation of the blood glucose levels in the 4 groups of rats (ODT, ACTOS^®^ (Commercial tablet), DM and Healthy) at time intervals of 1, 2, 4, 6, 12 and 24 h. Abbreviations: ODT, oral disintegrating tablet; DM, diabetic rats.

A significant reduction in blood glucose level was observed with ODT formulation as compared to the STZ diabetic control group (*p* < 0.05). Similarly, the commercial product (ACTOS^®^) attained a significant reduction (*p* < 0.05) in blood glucose level when it was compared to the diabetic control group (Figure 11). The maximum reduction in blood glucose level by ODT formulation was 350 mg/dL obtained after 4 h, while the commercial tablet attained maximum reduction in blood glucose level of 445 mg/dL after 6 h. However, ODT tablet performance was better than the commercial formulation in lowering blood glucose level (*p* > 0.05).

#### 3.5.4. Stability Studies

**Figure 12:**
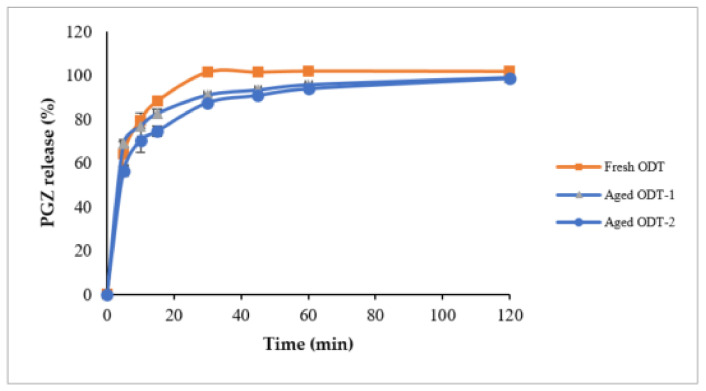
In vitro dissolution profile of Pioglitazone HCl from aged ODT tablets at accelerated storage condition; Aged ODT-1 at 25 ± 2 °C, 60 ± 5%; Aged ODT-2 at 40 ± 2 °C, 75 ± 5%. Abbreviations: ODT, orally disintegrating tablet; PGZ, Pioglitazone Hydrochloride.

It was shown that T-SNEDDS were able to keep >95% of intact PGZ after 3 months storage at 25 ± 2 °C, 60 ± 5% RH and 40 ± 2 °C, 75 ± 5% conditions (Figure 12). There was no significant difference among the release profiles of fresh and aged ODT formulations at both storage conditions (*p* > 0.05). All tested tablets showed no changes in their physical appearance.

## 4. Discussion

Self-nanoemulsifying formulations should be liquid monophasic systems of an oil-surfactant-cosurfactant mixture to present the drug in its solubilized form [33]. Therefore, the solubility is an important criterion to achieve high drug loading and prevent drug precipitation when introduced to GIT fluids [34,35]. PGZ structure (log *p* = 2.3) favors solubilization in small or medium molecular structures of fatty acids and/or mono- or diglycerides with their high ester content [36], amphiphilic surfactants and cosurfactants, rather than lipophilic structures and long-chain glycerides (Table 1).

During the development of L-SNEDDS, psuedoternary diagram data revealed that wider phase regions in system I > system II > system III indicated a better self-emulsification ability [37], this was attributed to the required hydrophilic lipophilic balance (RHLB) value; when the RHLB of the oil is similar or matches the corrected HLB for surfactant/cosurfactant blend, a stable emulsion with lowest interfacial tension is obtained [38]. RHLB values for Capryol 90 and Capmul MCM are 15 and 12.84, respectively [39]. For surfactant/cosurfactant blends, it is common to use one component with higher HLB (12–15), i.e., Cremophor RH40, Cremophor EL and Tween 80, with cosurfactants of low HLB (4–6), such as Transcutol HP and ethanol [40]. Consequently, a combination of a high HLB value surfactant Cremophor El (HLB = 14) with a low HLB value cosurfactant Transcutol (HLB = 4.2) was successful in forming SNEDDS with oil concentrations ranging from 10–50% *w*/*w* [41]. Moreover, a combination of a high HLB value surfactant Cremophor El (HLB = 14) with a high HLB value cosurfactant Propylene glycol (HLB = 11.5) was also successful, but with relatively with lower emulsification region at lower oil concentration ranges (10–40%). As a result of component synergism, a larger area of emulsification was obtained by using Cremophor EL/Transcutol as the surfactant blend when compared to the area resulting from systems that use Cremophor EL/PG as surfactant blends [38,39]. However, Cremophor EL (HLB = 14) was able to promote self-emulsification to yield emulsions with fine droplets that were expected be rapidly emptied from the stomach upon dispersion [42]. As reported by Basalious et al., Cremophor EL was one of the best surfactants with the highest emulsification efficiency requiring only one flask inversion for the formation of a homogenous emulsion [43].

Emulsion droplet size (PS) is an important index that reflects the rate and extent of drug release and consequently its absorption [44]. A good correlation was noticed between the oil content and the droplet size within the formulae. The droplet size increased when oil amount was increased [20]. However, there was no significant difference in droplets sizes when system I was compared to system II (*p* value > 0.05), where Capryol 90 was utilized as an oil phase. There was a marginal difference (*p* value < 0.05) in the droplet sizes in system III; Capmul MCM was utilized as the oil phase, the composition of the monoglycerides and free fatty acids contributed to the higher droplet size within the formulation [45]. In regard to particle size formulation stability and solubility, S14-iii was preferred over all L-SNEDDS formulations to blend a successful self-emulsifying formulation (Table 3 and Table 4).

For solidified SNEDDS, the slower release of PGZ from S-SNEDDS as compared to L-SNEDDS counterparts was attributed to the physical interaction of L-SNEDDS carrying the drug with the hydrophobic structures of silica particles within the adsorbent powder [46,47]. Upon adsorption, the lipid formulation becomes trapped in the intraparticular pores of the adsorbent particles and causes a reduction in the dissolution rate [48]. However, the difference in the release profiles among the different S-SNEDDS formulations was attributed to the structural differences among the four utilized silica powders; specific surface area of the powder controls the dissolution process and formulation release is higher with a larger surface area. The order of decreasing surface area was as follows: Syloid^®^ 244FP (380 m^2^/g) > Neusilin US2^®^ and Neusilin^®^ UFL2 (300 m^2^/g) > Aerosil^®^ PH200 (200 m^2^/g) [7,45]. Furthermore, it is important to mention that Aerosil (SiO_2_) is associated with more hydrophobic surface properties, which resulted in a decreased moisture uptake, as compared to the mesoporous structure of Mg–Al-silicates i.e., Syloid that have higher adsorption capacities and improved moisture uptake, that in turn improves the dissolution rate of the corresponding formulation [27,46]. Accordingly, SYL was preferred over the other types of adsorbents and was utilized in a ratio of 1:0.5 (L-SNEDDS: SYL) to produce S-SNEDDS powders with good flow properties. The overall characterization of S-SNEDDS powders confirmed that PGZ was present in its solubilized form within the S-SNEDDS formulation, and was protected from precipitation; this explains the loss of its crystallinity in DSC, XRD and FTIR patterns.

ODT formulation using Prosolv showed excellent tableting characteristics and enhancement of the PGZ release profile; the tablets disintegrated rapidly to release the self-nanoemulsifying formula where PGZ is solubilized upon exposure to GI fluids.

In vivo data revealed a significant improvement in PGZ pharmacokinetic profile in terms of C_max_ and AUC values for ODT formulation as compared to the raw drug suspension (*p* < 0.05). Moreover, the pharmacokinetic parameters obtained in the prepared ODT tablet were compared to the relevant commercially available film coated tablet (Actos^®^) and it was found that the prepared ODT formulation attained significant superior bioavailability (*p* < 0.05). Similarly, the correlated pharmacodynamic data showed a significant decrease in blood glucose levels with ODT formulation as compared to the commercially available tablet and raw drug suspension (*p* < 0.05). However, stability studies for ODT formulation showed no drug degradation or change in tablet characteristics.

## 5. Conclusions

Twelve PGZ liquid SNEDDS formulations were developed in this study. In that, the best formulation was selected with 20% Campul MCM, 20% Cremophore EL and 60% Propylene glycol, as an oil, a surfactant and a cosurfactant, respectively. The corresponding droplet size (121 nm) was less than 200 nm and a PDI value of 0.379 indicated the formation of emulsion with nanosized droplets and a uniform distribution. Moreover, it was stable against all phases of tested stability conditions and provided the maximum drug loading capacity. Solidification of the optimized L-SNEDDS into S-SNEDDS using SYL was successful in producing a freely flowing powder, free from drug interactions. However, the incorporation of S-SNEDDS into an ODT tablet was carried out using various tableting excipients. The selected ODT batch formulated with Prosolv was able to pass tablet quality control tests and stability tests. The ODT release profile was comparable to that of the marketed product and 2.5 folds higher than the raw drug. In vivo presentation of the optimized ODT batch showed evidence of improved bioavailability by higher C_max_ and AUC values than those of the commercially available tablet and raw drug suspension. The collective mechanism of self-nanoemulsifying drug delivery systems and orally disintegrating tablets led to improved solubility, stability, absorption and bioavailability of Pioglitazone Hydrochloride.

## Figures and Tables

**Table 1:** Equilibrium solubility values of Pioglitazone HCl (PGZ) in different oils, surfactants and cosolvents at 37 °C (mg/mL) ± SD *.

Component	Solubility (mg/mL ± SD)
Campul MCM	32.93 ± 0.826
Oleic acid	23.05 ± 0.286
Olive oil	16.4863 ± 0.437
Soybean oil	17.9653 ± 0.676
Caproyl 90	44.6897 ± 0.879
Cremophor^®^ RH40	9.2712 ± 0.970
Labrafil ^®^M1944CS	16.1616 ± 0.812
Labrasol	13.6003 ± 0.380
Tween ^®^80	35.533 ± 0.826
Cremophore EL	48.3765 ± 0.602
Propylene Glycol	249.99 ± 1.08
Transcutol^®^ HP	109.379 ± 0.687

* Data are the mean values (*n* = 3) ± SD.

**Table 2:** The composition of selected liquid self-nanoemulsifying formulations (L-SNEDDS).

System No.	Formula No.	Oil %	Surfactant %	Co- Surfactant %
(I) Oil: Capryol 90 Surfactant: Cremophore EL Cosurfactant: Transcutol HP	S14	20	20	60
	S18	40	30	30
	S19	30	30	40
	S21	10	30	60
(II) Oil: Capryol 90 Surfactant: Cremophore EL Cosurfactant: Propylene Glycol	S14	20	20	60
	S21	10	30	60
	S24	30	40	30
	S30	10	50	40
(III) Oil: Capmul MCM Surfactant: Cremophore EL Cosurfactant: Propylene Glycol	S14	20	20	60
	S23	40	40	20
	S24	30	40	30
	S30	10	50	40

**Table 3:** Emulsification time, percentage of transmittance (% T), droplet size (PS), polydispersity index (PDI), dispersibility grade, percentage of drug content and PGZ solubility in selected liquid self-nanoemulsifying formulations (L-SNEDDS) for systems I, II and III.

Formulation No.	Emulsification Time (sec ± SD)	% T	Particle Size (nm ± SD)	PDI	Grade	% Drug Content	Solubility (mg/g)
S14-i	34.06 ± 0.51	99.27%	36.85 ± 0.40	0.151	A	98.12 ± 2.89	18.47 ± 0.34
S18-i	49.67 ± 0.65	95.97%	61.82 ± 0.40	0.11	B	100.04 ± 1.67	21.57 ± 0.45
S19-i	45.78 ± 0.52	98.53%	48.97 ± 0.20	0.07	A	94.28 ± 1.67	32.68 ± 1.41
S21-i	29.29 ± 0.49	98.63%	40.21 ±0.85	0.56	A	92.35 ± 2.89	28.96 ± 0.27
S14-ii	31.24 ± 0.62	100.3%	22.1 ± 1.58	0.27	A	110.13 ± 0.15	177.12 ± 0.59
S21-ii	32.47 ± 0.32	98.93%	20.72 ± 0.57	0.271	A	98.92 ± 0.11	164.24 ± 0.16
S24-ii	60.53 ± 1.42	83.93%	52.97 ± 0.41	0.218	B	99.95 ± 0.43	69.76 ± 0.63
S30-ii	49.83 ±0.30	100.1%	15.58 ± 0.48	0.185	A	99.18 ± 0.18	127.74 ± 0.54
S14-iii	15.25 ± 0.80	96%	121.97 ± 1.02	0.379	A	99.03 ± 0.41	202.56 ± 0.63
S23-iii	29.18 ± 0.90	90.4%	349.73 ± 11.37	0.421	B	98.48 ± 0.36	135.82 ± 1.89
S24-iii	21.35 ± 0.91	93.5%	124.2 ± 13.8	0.362	A	95.95 ± 1.9	186.18 ± 0.49
S30-iii	19.75 ± 0.61	99.9%	14.12 ± 0.06	0.053	A	94.52 ± 1.44	191.85 ± 0.62

Abbreviations: % T, percentage of transmittance; PDI, polydispersity index; S-i, S-ii and S-iii, formulations related to systems I, II and III respectively etc.

**Table 4:** Thermodynamic stability tests for selected liquid self-nanoemulsifying formulations (L-SNEDDS).

Formulation No.	Observations Based on Thermodynamic Stability Tests		
	H/C	Cent	F/T
S14-i	✓	✓	✓
S18-i	✓	✓	✕
S19-i	✕	✕	✕
S21-i	✓	✓	✕
S14-ii	✓	✓	✓
S21-ii	✓	✓	✓
S24-ii	✓	✓	✕
S30-ii	✓	✓	✓
S14-iii	✓	✓	✓
S23-iii	✓	✕	✕
S24-iii	✓	✓	✓
S30-iii	✓	✓	✓

Abbreviations: H/C, heating cooling cycles; Cent, centrifugation; F/T, freeze/thaw cycles.

**Table 5:** Components of solidified PGZ SNEDDS (S-SNEDDS) formulations using different adsorbent types.

Formulation No.	L-SNEDDS Composition	Adsorbent Type	(L-SNEDD: Adsorbent)
S-SNEDDS-1	15 mg PGZ in 1 g S14-iii (20% Capmul MCM, 20% Cremophor EL and 60% Propylene Glycol)	Neusilin^®^ US2	1:0.5
S-SNEDDS-2		Neusilin^®^ UFL2	1:0.5
S-SNEDDS-3		Syloid^®^ 244FP	1:0.5
S-SNEDDS-4		Aerosil^®^ PH200	1:0.5

Abbreviations: L-SNEDDS, liquid self-nanoemulsifying drug delivery system; S-SNEDDS, solidified self-nanoemulsifying drug delivery system.

**Table 6:** Effect of L-SNEDDS: Syloid (SYL) ratio on the flowability of S-SNEDDS powder.

Formula No.	Ratio (L-SNEDDS: SYL)	Appearance	ρ_bulk_	ρ_tap_	HR	CI	θ	Flowability
R1	1:0.25	Caking and Wet	0.13	0.18	1.38	27	50.2	Poor
R2	1:0.5	Fine and Dry	0.17	0.20	1.18	15	35.3	Good
R3	1:1	Fine and Dry	0.12	0.14	1.16	14.28	36.7	Good
R4	1:2	Dusty	0.085	0.09	1.05	5.56	27.7	Excellent

Abbreviations: ρbulk, ρtap, bulk and tapped densities; HR, Hausner ratio; CI, compressibility index; θ, angle of repose; L-SNEDDS, liquid self-nanoemulsifying drug delivery system; S-SNEDDS, solidified self-nanoemulsifying drug delivery system; SYL, Syloid.

**Table 7:** Composition of prepared ODT tablets.

Formulation	% Composition		Weight (mg)
L-SNEDDS	Capmul MCM 20%, Cremophor EL 20%, PG 60%		100 mg
S-SNEDSS	L-SNEDDS: SYL (1:0.5)		50 mg
ODT excipients	Mannitol, Acdisol, lactose, Prosolv, Mg stearate		~350 mg
Ingredients	ODT-1	ODT-2	ODT-3
Drug incorporated in S-SNEDDS	110	110	110
Lactose	-	335	-
Mannitol	-	-	335
Acdisol	-	50	50
Prosolv	385	-	-
Mg stearate	5	5	5
Total Weight (mg)	500	500	500

Abbreviations: ODT, orally disintegrating tablets; L-SNEDDS, liquid self-nanoemulsifying drug delivery system; S-SNEDDS, solidified self-nanoemulsifying drug delivery system; SYL, Syloid.

**Table 8:** Quality parameters of PGZ orally disintegrating tablets (ODT).

Parameter	*n* *	Results
Diameter (mm)	10	13.41± 0.01
Thickness (mm)	10	3.08 ± 0.01
Weight Variation (%)	10	0.042%
Content Uniformity %	10	99.92% ± 1.2
Friability %	13	0.68%
Hardness (kp)	6	5.73 ± 0.63
Disintegration Time (sec)	6	28.36 ± 0.95

* Number of tablets used to perform the test.

**Table 9:** The plasma pharmacokinetic parameters of the ODT tablet, commercial tablet and raw PGZ after a single dose oral administration to Wistar rats (mean ± SD, *n* = 6).

Pharmacokinetic Parameter	ODT	ACTOS	Raw PGZ
C_max_ (µg/mL)	27.77 ± 2.55	17.40 ± 3.38	12.91 ± 2.24
T_max_ (h)	3.4 ± 1.14	3.0 ± 1.00	3.0 ± 0.89
AUC_0−∞_ (µgh/mL)	313.05	185.32	151.36
AUMC_0−∞_ (µg h^2^/^mL^)	3461.02	1738.71	1652.14
K_el_ (1/hr)	0.18 ± 0.082	0.117 ± 0.013	0.126 ± 0.05
t*_1/2_*(h)	5.57 ± 4.94	5.95 ± 0.719	6.85 ± 4.35
MRT (h)	7.19 ± 0.96	7.87 ± 0.59	7.725 ± 0.84

Abbreviations: ODT, oral disintegrating tablet; PGZ, Pioglitazone Hydrochloride; C_max_: maximum plasma concentration; t_max_: time to C_max_; AUC_0−∞_: total area under the concentration-time curve; AUMC_0−∞_: total area under the first moment curve; K_el_, elimination rate constant; t*_1/2_*: elimination half-life; MRT, mean residence time.

## Data Availability

Not applicable.

## References

[1] Scientific Discussion, European Medicines Agency. www.emeuropeasia.org.

[2] Charman S.A., Charman W.N., Rogge M.C., Wilson T.D., Dutko F.J., Pouton C.W. (1992). Self-emulsifying drug delivery systems: Formulation and biopharmaceutic evaluation of an investigational lipophilic compound. Pharm. Res..

[3] Porter C.J.H., Pouton C.W., Cuine J.F., Charman W.N. (2008). Enhancing intestinal drug solubilisation using lipid-based delivery systems. Adv. Drug Deliv. Rev..

[4] Lei Y., Lu Y., Qi J., Nie S., Hu F., Pan W., Wu W. (2011). Solid self-nanoemulsifying cyclosporin A pellets prepared by fluid-bed coating: Preparation, characterization and in vitro redispersibility. Int. J. Nanomed..

[5] Gumaste S.G., Dalrymple D.M., Serajuddin A.T. (2013). Development of solid SEDDS, V: Compaction and drug release properties of tablets prepared by adsorbing lipid-based formulations onto Neusilin^®^ US2. Pharm. Res..

[6] Shahba A.A.-W., Ahmed A.R., Alanazi F.K., Mohsin K., Abdel-Rahman S.I. (2018). Multi-layer self-nanoemulsifying pellets: An innovative drug delivery system for the poorly water-soluble drug cinnarizine. AAPS Pharmscitech.

[7] Khanfar M., Al-Nimry S. (2017). Stabilization and amorphization of lovastatin using different types of silica. AAPS PharmSciTech.

[8] Abd-Elhakeem E., Teaima M.H., Abdelbary G.A., El Mahrouk G.M. (2019). Bioavailability enhanced clopidogrel-loaded solid SNEDDS: Development and in-vitro/in-vivo characterization. J. Drug Deliv. Sci. Technol..

[9] Nguyen T.H., Tan A., Santos L., Ngo D., Edwards G.A., Porter C.J., Prestidge C.A., Boyd B.J. (2013). Silica–lipid hybrid (SLH) formulations enhance the oral bioavailability and efficacy of celecoxib: An in vivo evaluation. J. Control. Release.

[10] Midha K., Nagpal M., Aggarwal G., Singh T.G. (2015). Development of dispersible self-microemulsifying tablet of atorvastatin. Pharm. Methods.

[11] Asthana A., Aggarwal S., Asthana G. (2013). Oral dispersible tablets: Novel technology and development. Int. J. Pharm. Sci. Rev. Res..

[12] Higuchi T. (1965). A phase solubility technique. Adv. Anal. Chem. Instrum..

[13] El-Laithy H.M., Basalious E.B., El-Hoseiny B.M., Adel M.M. (2015). Novel self-nanoemulsifying self-nanosuspension (SNESNS) for enhancing oral bioavailability of diacerein: Simultaneous portal blood absorption and lymphatic delivery. Int. J. Pharm..

[14] Sjöholm E., Sandler N. (2019). Additive manufacturing of personalized orodispersible warfarin films. Int. J. Pharm..

[15] Zhang P., Liu Y., Feng N., Xu J. (2008). Preparation and evaluation of self-microemulsifying drug delivery system of oridonin. Int. J. Pharm..

[16] Ammar H., Ghorab M., Mostafa D.M., Ghoneim A.M. (2013). Self-nanoemulsifying drug delivary system for sertraline hydrochloride: Design, preparation and characterization. Future.

[17] Yin Y.M., Cui F.D., Mu C.F., Choi M.K., Kim J.S., Chung S.J., Shim C.K., Kim D.D. (2009). Docetaxel microemulsion for enhanced oral bioavailability: Preparation and in vitro and in vivo evaluation. J. Control. Release.

[18] Mohd A.B., Sanka K., Bandi S., Diwan P.V., Shastri N. (2015). Solid self-nanoemulsifying drug delivery system (S-SNEDDS) for oral delivery of glimepiride: Development and antidiabetic activity in albino rabbits. Drug Deliv..

[19] U.S. Food and Drug Database. www.accessdata.fda.gov.

[20] Shafiq S., Shakeel F., Talegaonkar S., Ahmad F.J., Khar R.K., Ali M. (2007). Development and bioavailability assessment of ramipril nanoemulsion formulation. Eur. J. Pharm. Biopharm..

[21] Pathade P., Imran M., Bairagi V., Ahire Y. (2011). Development and validation of stability indicating UV spectrophotometric method for the estimation of sitagliptin phosphate in bulk and tablet dosage form. J. Pharm. Res..

[22] Zhang Y., Huo M., Zhou J., Xie S. (2010). PKSolver: An add-in program for pharmacokinetic and pharmacodynamic data analysis in Microsoft Excel. Comput. Methods Programs Biomed..

[23] The ICH Guidelines for Stability Testing of Active Pharmaceutical Ingredients and Finished Pharmaceutical Products (Annex10). https://database.ich.org/sites/default/files/Q1F_Stability_Guideline_WHO_2018.pdf.

[24] Wang L., Dong J., Chen J., Eastoe J., Li X. (2009). Design and optimization of a new self-nanoemulsifying drug delivery system. J. Colloid Interface Sci..

[25] Madan J.R., Sudarshan B., Kadam V.S., Kama D. (2014). Formulation and development of self-microemulsifying drug delivery system of pioglitazone. Asian J. Pharm..

[26] Mohsin K., Long M.A., Pouton C.W. (2009). Design of lipid-based formulations for oral administration of poorly water-soluble drugs: Precipitation of drug after dispersion of formulations in aqueous solution. J. Pharm. Sci..

[27] Shahba A., Ahmed A.R., Mohsin K., Abdel-Rahman S.I., Alanazi F.K. (2017). Solidification of cinnarizine self-nanoemulsifying drug delivery systems by fluid bed coating: Optimization of the process and formulation variables. Pharm. Int. J. Pharm. Sci..

[28] Al-Khattawi A., Mohammed A.R. (2013). Compressed orally disintegrating tablets: Excipients evolution and formulation strategies. Expert Opin. Drug Deliv..

[29] Manivannan R. (2009). Oral disintegrating tablets: A future compaction. Drug Invent. Today.

[30] Dobetti L. (2001). Fast-melting tablets: Developments and technologies. Pharm. Technol..

[31] Guidance for Industry: Orally Disintegrating Tablets (2008). FDA CDER.

[32] Deshmukh A., Kulkarni S. (2014). Solid self-microemulsifying drug delivery system of ritonavir. Drug Dev. Ind. Pharm..

[33] Basalious E.B., Shawky N., Badr-Eldin S.M. (2010). SNEDDS containing bioenhancers for improvement of dissolution and oral absorption of lacidipine. I: Development and optimization. Int. J. Pharm..

[34] Kommuru T., Gurley B., Khan M., Reddy I. (2001). Self-emulsifying drug delivery systems (SEDDS) of coenzyme Q10: Formulation development and bioavailability assessment. Int. J. Pharm..

[35] Elnaggar Y.S., El-Massik M.A., Abdallah O.Y. (2009). Self-nanoemulsifying drug delivery systems of tamoxifen citrate: Design and optimization. Int. J. Pharm..

[36] Rane S.S., Anderson B.D. (2008). What determines drug solubility in lipid vehicles: Is it predictable?. Adv. Drug Deliv. Rev..

[37] Smail S.S., Ghareeb M.M., Omer H.K., Al-Kinani A.A., Alany R.G. (2021). Studies on surfactants, cosurfactants, and oils for prospective use in formulation of ketorolac tromethamine ophthalmic nanoemulsions. Pharmaceutics.

[38] Rao M.R., Aghav S., Sukre G., Kumar M. (2014). Determination of required HLB of Capryol 90. J. Dispers. Sci. Technol..

[39] Legen I., Peternel L., Novak S., Homar M., Rozman P., Klancar U. (2013). Self-Microemulsifying Drug Delivery System of Abiraterone or Abiraterone. Acetate. Patent.

[40] Kang B.K., Lee J.S., Chon S.K., Jeong S.Y., Yuk S.H., Khang G., Lee H.B., Cho S.H. (2004). Development of self-microemulsifying drug delivery systems (SMEDDS) for oral bioavailability enhancement of simvastatin in beagle dogs. Int. J. Pharm..

[41] Eid A.M.M., Baie S.H., Arafat O.M. (2012). The effect of surfactant blends on the production of self-emulsifying system. Int. J. Pharm. Front. Res..

[42] Constantinides P.P., Scalart J.P., Lancaster C., Marcello J., Marks G., Ellens H., Smith P.L. (1994). Formulation and intestinal absorption enhancement evaluation of water-in-oil microemulsions incorporating medium-chain glycerides. Pharm. Res..

[43] Alhasani K.F., Kazi M., Ibrahim M.A., Shahba A.A., Alanazi F.K. (2019). Self-nanoemulsifying ramipril tablets: A novel delivery system for the enhancement of drug dissolution and stability. Int. J. Nanomed..

[44] Ghosh D., Singh S.K., Khursheed R., Pandey N.K., Kumar B., Kumar R., Kumari Y., Kaur G., Clarisse A., Awasthi A. (2020). Impact of solidification on micromeritic properties and dissolution rate of self-nanoemulsifying delivery system loaded with docosahexaenoic acid. Drug Dev. Ind. Pharm..

[45] Verma S., Singh S.K., Verma P.R.P. (2016). Solidified SNEDDS of loratadine: Formulation using hydrophilic and hydrophobic grades of Aerosil®, pharmacokinetic evaluations and in vivo–in silico predictions using GastroPlus™. RSC Adv..

[46] Hespeler D., El Nomeiri S., Kaltenbach J., Müller R.H. (2019). Nanoporous smartPearls for dermal application–Identification of optimal silica types and a scalable production process as prerequisites for marketed products. Beilstein J. Nanotechnol..

[47] Choudhari Y., Reddy U., Monsuur F., Pauly T., Hoefer H., McCarthy W. (2014). Comparative evaluation of porous silica-based carriers for lipids and liquid drug formulations. Open Mater. Sci..

[48] Kharechkina E.S., Nikiforova A.B., Belosludtsev K.N., Rokitskaya T.I., Antonenko Y.N., Kruglov A.G. (2021). Pioglitazone Is a Mild Carrier-Dependent Uncoupler of Oxidative Phosphorylation and a Modulator of Mitochondrial Permeability Transition. Pharmaceuticals.

